# Facial Melanosis in Skin of Color: An Integrated Clinical, Dermoscopic, and Quality of Life Analysis

**DOI:** 10.7759/cureus.102816

**Published:** 2026-02-02

**Authors:** Aniket Goswami, Anita Marak, Shikha Verma, Bikash Chingshubam, Saqib Raza Hasan Khan

**Affiliations:** 1 Dermatology, North Eastern Indira Gandhi Regional Institute of Health and Medical Sciences, Shillong, IND

**Keywords:** dermoscopy, facial melanosis, melasma, quality of life, skin of color

## Abstract

Background: Facial melanosis comprises a heterogeneous spectrum of pigmentary disorders that are particularly prevalent and persistent in skin of color. Despite its frequency, comprehensive data integrating clinical patterns, dermoscopic features, and quality of life impact remain limited, especially from Northeast India.

Objectives: This study aimed to characterize the clinical spectrum of facial melanosis, delineate dermoscopic patterns, including atypical features, and evaluate health-related quality of life using Skindex-16 in a skin of color population.

Materials and methods: This hospital-based cross-sectional study included 150 untreated adults with facial melanosis. Participants underwent detailed clinical evaluation, dermoscopic assessment using a polarized video dermoscope, and quality of life assessment with Skindex-16. Diagnoses were classified as standalone or overlapping entities.

Results: Twenty-two clinical conditions were identified, with melasma being the most prevalent diagnosis (n = 102, 68.0%), showing a marked female preponderance. Overlapping presentations, particularly melasma with topical steroid damaged face (TSDF), were common. Dermoscopy revealed predominant pigmentary patterns such as brown structureless areas and globules, alongside vascular and appendageal changes. Importantly, infrequent atypical dermoscopic features, rarely reported in facial melanosis, were documented. Quality of life analysis demonstrated a disproportionate emotional burden despite relatively low symptom scores.

Conclusion: Facial melanosis in skin of color is clinically complex, frequently overlapping, and psychosocially impactful. The integration of dermoscopy and quality of life assessment provides a nuanced, patient-centered understanding, with the identification of atypical dermoscopic features representing a distinctive contribution of this study.

## Introduction

Facial melanosis describes a continuum of facial pigmentary disorders in which disturbances of melanocyte function, shaped by sunlight, inflammation, hormones, and heritage, manifest visibly on the most expressive surface of the human body [1,2]. In skin of color, where eumelanin is both sentinel and storyteller, pigmentary change often becomes more than an alteration of chromatic balance; it reflects the intimate convergence of biological predisposition and environmental influence.

This spectrum includes not only melasma and post-inflammatory hyperpigmentation (PIH) but also entities such as lichen planus pigmentosus (LPP), Riehl’s melanosis, Hori’s nevus, periorbital melanosis, pigmentary demarcation lines, and the distinctive compound patterns frequently seen in darker phototypes [1,2]. These entities share a foundational biology, which includes heightened melanocyte reactivity, larger melanosomes, and more efficient melanin transfer that amplify even subtle provocations [3]. Ultraviolet and visible light exposures, cosmetic practices, hormonal fluctuations, and genetic architecture collectively shape their clinical variability, while inflammatory or exogenous insults determine chronicity and depth [4].

Yet the burden of facial pigmentation is not solely cutaneous. The face serves as a social interface, and alterations in its tonal harmony may evoke emotional distress, altered self-perception, and functional impairment. Instruments such as Skindex-16 help quantify these internal reverberations by capturing symptoms, emotions, and functional impact, underscoring that pigment is as much a psychological phenomenon as a biological one [5].

Despite its prevalence in skin of color, pigmentary pathology remains underrepresented in global literature. This contributes to diagnostic uncertainty, insufficient photoprotection guidance, and limited treatment safety data, particularly concerning lasers and chemical procedures in richly pigmented skin. Recent expert consensus underscores critical gaps in skin of color-specific research and advocates for contextually informed, ethno-dermatologic perspectives [6].

Within this landscape, Northeast India, marked by ethnic diversity, a high ultraviolet index, and distinct cultural practices, remains inadequately studied. Characterizing its clinical and dermoscopic patterns of facial melanosis, alongside quality of life impact, offers essential insights into both the biology and lived experience of pigment in skin of color. 

Accordingly, the objectives of this study were to characterize the clinical spectrum of facial melanosis, delineate dermoscopic patterns, including atypical features, and evaluate health-related quality of life using Skindex-16 in a skin of color population.

## Materials and methods

Study design and setting

This was a hospital-based, cross-sectional descriptive study conducted in the department of dermatology at the North Eastern Indira Gandhi Regional Institute of Health and Medical Sciences, Shillong, India, catering to the diverse population of northeast India, over a one-year period from June 2024 to May 2025.

Study population

All eligible participants encountered during the study period were recruited using consecutive sampling until the end of the study period.

Inclusion criteria

All adult patients with a clinical diagnosis of facial hyperpigmentation, irrespective of etiology were included in the study.

Exclusion criteria

Any patient who underwent treatment for facial hyperpigmentation within the preceding four weeks was excluded.

Ethical approval

The study protocol was reviewed and approved by the institutional ethics committee of North Eastern Indira Gandhi Regional Institute of Health and Medical Sciences, Shillong (approval number: NEIGR/IECIM1/T4/2024), and written informed consent was obtained from all participants prior to enrollment.

Clinical evaluation and data collection

Each participant underwent a structured clinical evaluation, documented using a predesigned case record form.

Wood’s lamp examination

Wood’s lamp examination was performed in a darkened room using a stationary Wood’s lamp emitting ultraviolet light at approximately 365 nm. Pigmentation was assessed for accentuation, which helped in estimating the depth of pigment as epidermal (accentuation), dermal (no accentuation), or mixed pattern.

Dermoscopic assessment

Dermoscopic evaluation was performed using a video dermoscope (DinoLite-Pro, AnMo Electronics Corporation, Taiwan, Republic of China) with a polarized lens and integrated light-emitting diode (LED) illumination.

Procedure

The procedure involved cleaning the lesion under focus with a 70% isopropyl alcohol swab, following which the dermoscope was applied in direct contact with the lesion, and the parameters assessed included background color and pigmentation, pigment patterns, vascular patterns, and appendageal patterns.

Patterns were interpreted in accordance with previously described dermoscopic criteria for facial melanoses.

Quality of life assessment

Health-related quality of life was assessed using the Skindex-16 questionnaire (English version) after obtaining permission from MAPI Research Trust (User License No. 91285) [5].

The questionnaire comprised 16 items across three domains: symptoms, emotions, and functioning (Appendix A). Responses were recorded on a seven-point Likert scale and transformed to a 0-100 linear scale, with higher scores indicating greater impairment. Domain scores and composite scores were calculated as per standard guidelines, with predefined handling of missing data.

Statistical analysis 

The data were entered in Microsoft Excel for Mac Version 16.98 (Microsoft Corp., Redmond, WA, USA) and analyzed using jamovi software (version 2.6.25.0, retrieved from https://www.jamovi.org) [7]. Categorical variables were expressed as frequencies and percentages, while continuous variables were summarized as mean with standard deviation or median with interquartile range (IQR), as appropriate.

## Results

Demographic profile of the study participants

During the study period, 258 of 8,608 newly registered dermatology patients (~3.0%) were diagnosed with facial melanosis at the study center. After excluding individuals who had received treatment for facial pigmentation within the preceding four weeks (n = 108), a total of 150 untreated participants were included in the final analysis.

The mean age was 38.2 ± 10.0 years (range: 19-70 years), with a median age of 37.0 years (IQR: 30.0-44.8 years). There was a marked female predominance, with 122 females (81.3%) and 28 males (18.7%), corresponding to a male-to-female ratio of 0.23. All the participants either had a Fitzpatrick phototype of type IV or V. The socio-economic status was assessed using the modified Kuppuswamy socioeconomic scale [8]. The socio-demographic details of the participants are summarized in Table 1.

**Table 1:** Socio-Demographic Profile of Study Participants (N = 150) *Service sector includes office work, education, healthcare, government service, defence, and religious services; †Assessed using the Modified Kuppuswamy Socioeconomic Scale [8].

Variable	Category	N	Percentage (%)
Educational attainment	Undergraduate / Postgraduate	89	59.3
	Secondary (High school / Matriculation)	38	25.3
	Primary / Middle school	14	9.3
	Professional / Vocational	9	6.0
Occupation	Homemaker	56	37.3
	Service sector*	41	27.3
	Self-employed / Business	21	14.0
	Student	17	11.3
	Skilled / Unskilled / Technical	15	10.0
Socioeconomic status†	Lower middle	82	54.7
	Upper lower	45	30.0
	Upper middle	22	14.7
	Lower	1	0.7
Ethnicity	Bengali	37	24.7
	Khasi	36	24.0
	Assamese	28	18.7
	Bihari	11	7.3
	Nepali	6	4.0
	Jaintia	6	4.0
	Mizo	5	3.3
	Garo	4	2.7
	Nagamese	4	2.7
	Manipuri	3	2.0
	Marwari	3	2.0
	Tripuri	3	2.0
	Apatani	2	1.3
	Aka	1	0.7
	Mishmi	1	0.7
Fitzpatrick skin phototype	Type IV	76	50.7
	Type V	74	49.3

Clinical spectrum of facial melanosis

**Figure 1:**
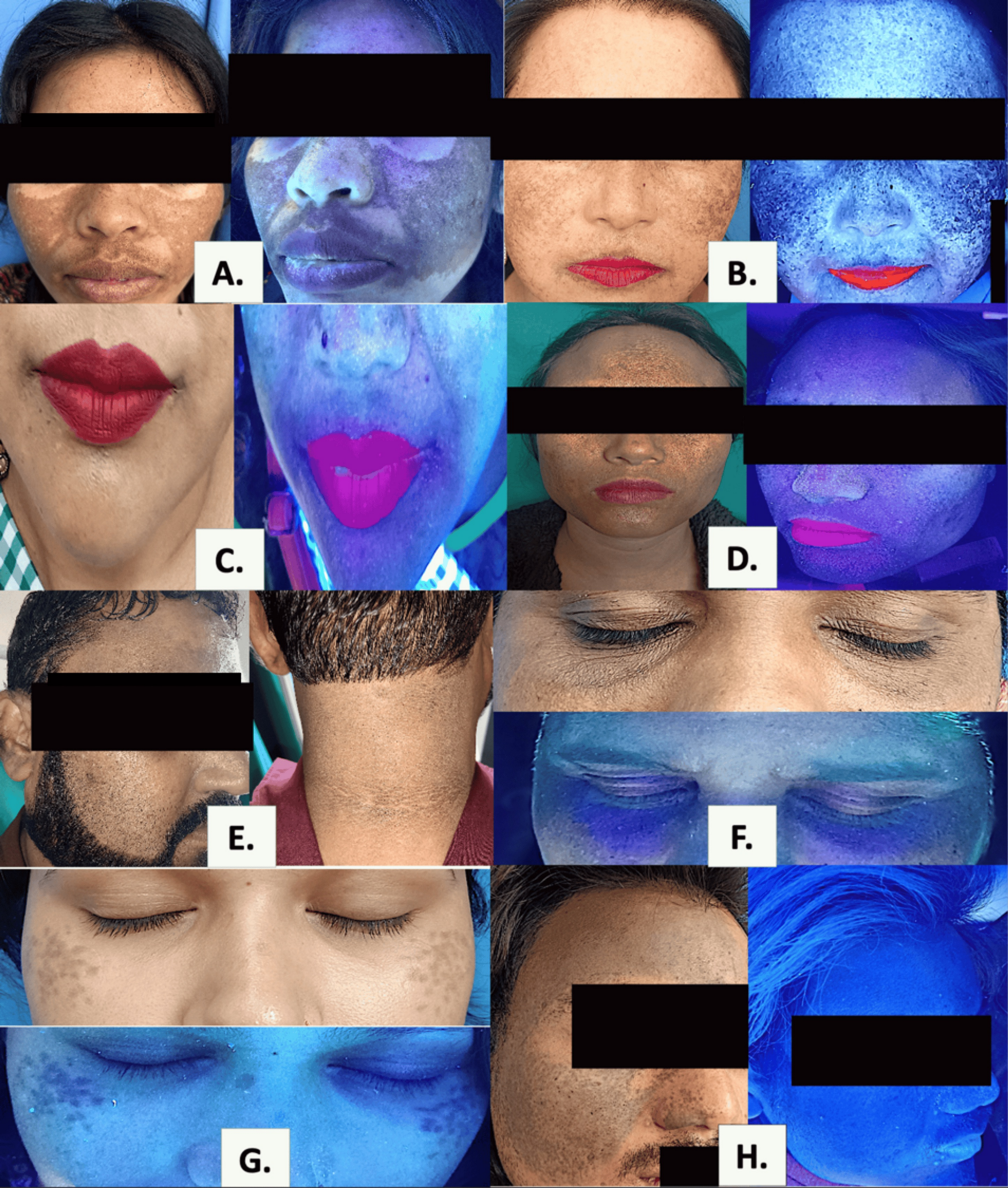
Clinical and Wood’s Lamp Images of Various Facial Melanosis A. Melasma; B. Melasma with photoageing; C. Erythrosis pigmentosa peribuccalis of Brocq; D. Lichen planus pigmentosus; E. Acanthosis nigricans; F. Periorbital hyperpigmentation; G.Hori's nevus; H. Nevus of Ota

**Figure 2:**
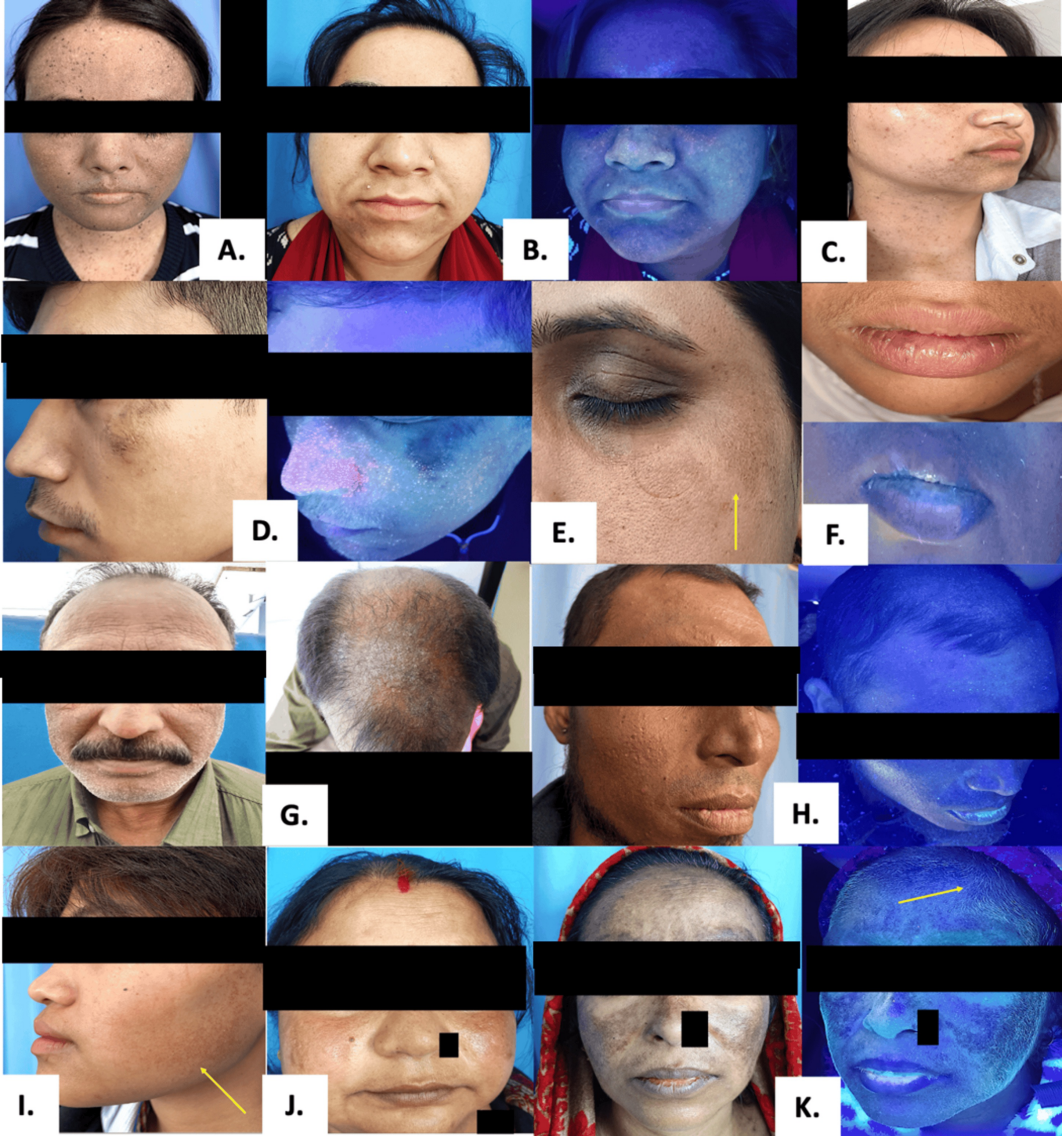
Clinical and Wood’s Lamp Images of Various Facial Melanosis A. Xeroderma pigmentosum; B. Seborrhoeic melanosis; C. Unilateral partial lentiginosis; D. Exogenous traumatic tattooing; E. Type G pigmentary demarcation lines (yellow arrow); F. Mucosal lentigines; G. Riehl's melanosis; H. Chronic liver disease induced pigmentation; I. Erythromelanosis follicularis faciei et colli; J. Topical steroid-damaged face; K. Topical steroid-damaged face with leucotrichia (yellow arrow)

A total of 22 broader clinical categories were identified in the study population (Table 2). Owing to the frequent coexistence of multiple pigmentary disorders within individual participants, these categories manifested clinically as 37 distinct diagnostic entities, representing specific permutations of disease overlap. These entities comprised 21 standalone presentations, in which a single condition occurred in isolation, and 16 overlapping presentations, defined by the co-occurrence of two or more pigmentary disorders. Consequently, while the disease spectrum was structured around 22 clinical categories, their varied combinations resulted in a larger number of observable diagnostic patterns (Figure 1 and Figure 2), and category-wise frequencies were not mutually exclusive, with cumulative counts exceeding the total sample size (N = 150) by design.

**Table 2:** Distribution of Broader Clinical Categories of Facial Melanosis in the Study Population (N = 150) * Individual counts represent the number of participants exhibiting the specified clinical category, either as a standalone diagnosis or as part of an overlapping presentation. ** Percentages are calculated out of the total study population (N = 150); categories are not mutually exclusive.

No.	Broader Clinical Category	Individual Counts*	Proportion (%)**
1	Melasma	102	68.0
2	Topical steroid-damaged face (TSDF)	39	26.0
3	Lentigines	15	10.0
4	Lichen planus pigmentosus (LPP)	11	7.3
5	Erythrosis pigmentosa peribuccalis of Brocq (EPPB)	11	7.3
6	Periorbital hyperpigmentation (POH)	9	6.0
7	Pigmentary demarcation lines (PDL)	7	4.7
8	Nevus of Ota	6	4.0
9	Post-inflammatory hyperpigmentation (PIH)	5	3.3
10	Ashy dermatosis	4	2.7
11	Acanthosis nigricans (AN)	3	2.0
12	Riehl’s melanosis	3	2.0
13	Photoageing	3	2.0
14	Hori’s nevus	3	2.0
15	Mucosal lentigines	1	0.7
16	Exogenous pigmentation / accidental tattooing	1	0.7
17	Nevus spilus	1	0.7
18	Erythromelanosis follicularis faciei et colli (EMFFEC)	1	0.7
19	Chronic liver disease (CLD) induced pigmentation	1	0.7
20	Seborrhoeic melanosis (SM)	1	0.7
21	Unilateral partial lentiginosis	1	0.7
22	Xeroderma pigmentosum (XP)	1	0.7

Standalone presentations

Among standalone diagnoses, melasma was the most prevalent (n = 37, 24.7%), followed by LPP (n = 9, 6.0%), nevus of Ota (n = 6, 4.0%), ashy dermatosis (n = 4, 2.7%), and Riehl's melanosis and Hori's nevus (each n = 3, 2.0%). Acanthosis nigricans (AN) (n = 3, 2.0%) and periorbital hyperpigmentation (POH) (n = 2, 1.3%) were also observed. Less frequent standalone conditions included various forms of PIH, such as PIH secondary to varicella, fixed drug eruption, systemic lupus erythematosus (SLE), and trauma (each ranging from n = 1-2, 0.7-1.3%). Singular cases were also recorded for mucosal lentigines, nevus spilus, chronic liver disease (CLD)-induced pigmentation, exogenous pigmentary changes (e.g., accidental tattooing), erythromelanosis follicularis faciei et colli (EMFFEC), seborrheic melanosis (SM), unilateral partial lentiginosis, xeroderma pigmentosum (XP), and solitary lentigines (each n = 1, 0.7%).

Overlapping presentations

The most frequent among these was melasma with topical steroid-damaged face (TSDF), documented in 30 patients (20.0%). Other common combinations included melasma with erythrosis pigmentosa peribuccalis of Brocq (EPPB; n = 9, 6.0%) and melasma with lentigines (n = 9, 6.0%), followed by melasma with POH (n = 4, 2.7%) and melasma with PDL (pigmentary demarcation lines) (n = 3, 2.0%). Three cases (2.0%) involved melasma with both TSDF and lentigines. Additional rare overlapping constellations included combinations with photoageing, LPP, EPPB, and PDL. Unique dual or triple associations such as melasma, LPP, and TSDF; melasma, lentigines, EPPB, and TSDF; PDL with POH; and POH with TSDF were each noted in single individuals (n = 1, 0.7%). Among the seven participants (4.7%) identified with pigmentary demarcation lines, Type F was the most common subtype (n = 4), followed by Type H (n = 1), while one participant exhibited a composite Type G + H pattern.

Clinically, brown pigmentation was predominant (n = 96, 64.0%), followed by grey pigmentation (n = 33, 22.0%), mixed hues (n = 17, 11.3%), and bluish-grey pigmentation (n = 4, 2.7%). Pigmentation was diffuse in pattern in 108 participants (72.0%) and symmetrical in 131 participants (87.3%). Extra-facial pigmentation was documented in 18 participants (12.0%), most commonly involving the neck, particularly in LPP, AN, and ashy dermatosis.

Duration of facial pigmentation and sun exposure

The median duration was 3.0 years (IQR 1.0-5.0 years). The duration of disease varied widely, ranging from approximately one month (0.08 years) to 20.0 years. The median daily sun exposure among participants was 1.0 hour/day (IQR: 2.0 hours).

Cosmetic and topical product use

Use of cosmetic or topical products was reported by 91 participants (60.7%). Among those reporting product use, fairness creams were the most frequently used (n = 14, 15.4%), followed by anti-ageing creams (n = 11, 12.1%). The majority, however, reported use of non-specific topical agents, including moisturisers, multipurpose emollients, and oils such as coconut or mustard oil (n = 65, 71.4%). A small proportion reported hair dye use (n = 1, 1.1%).

History of medication use

A past history of medication (prior to four weeks) use relevant to facial pigmentation was reported by 32 participants (21.3%). Topical corticosteroids constituted the most frequently reported agents, used by 29 participants (90.6%) as fixed-dose combination formulations, commonly containing corticosteroids with antibiotics, antifungals, or keratolytic agents. Non-corticosteroid medications were reported by three participants (9.4%) and included oral fluconazole, hydroxychloroquine, and a combination of rifaximin with propranolol, each reported by one participant (3.1%).

Hormonal and reproductive factors

Among female participants, pregnancy-related exacerbation of pigmentation was reported by 20 participants (13.3%), all of whom were diagnosed with melasma. Use of oral contraceptive pills was reported by 13 female participants (8.7%), again exclusively among those with melasma.

Associated factors

Smoking was reported by 17 participants (11.3%) and alcohol consumption by 31 (20.7%). Comorbidities were present in 30 participants (20.0%), chiefly hypothyroidism (n = 16), exclusively in patients with melasma. A positive family history was reported by 32 participants (21.3%), predominantly involving first-degree female relatives, most commonly noted in those with melasma.

Symptom profile

Itching was the most common symptom (n = 41, 27.3%), followed by burning sensation (n = 20, 13.3%) and pain (n = 5, 3.3%), predominantly in participants with TSDF.

Dermoscopic findings

**Figure 3:**
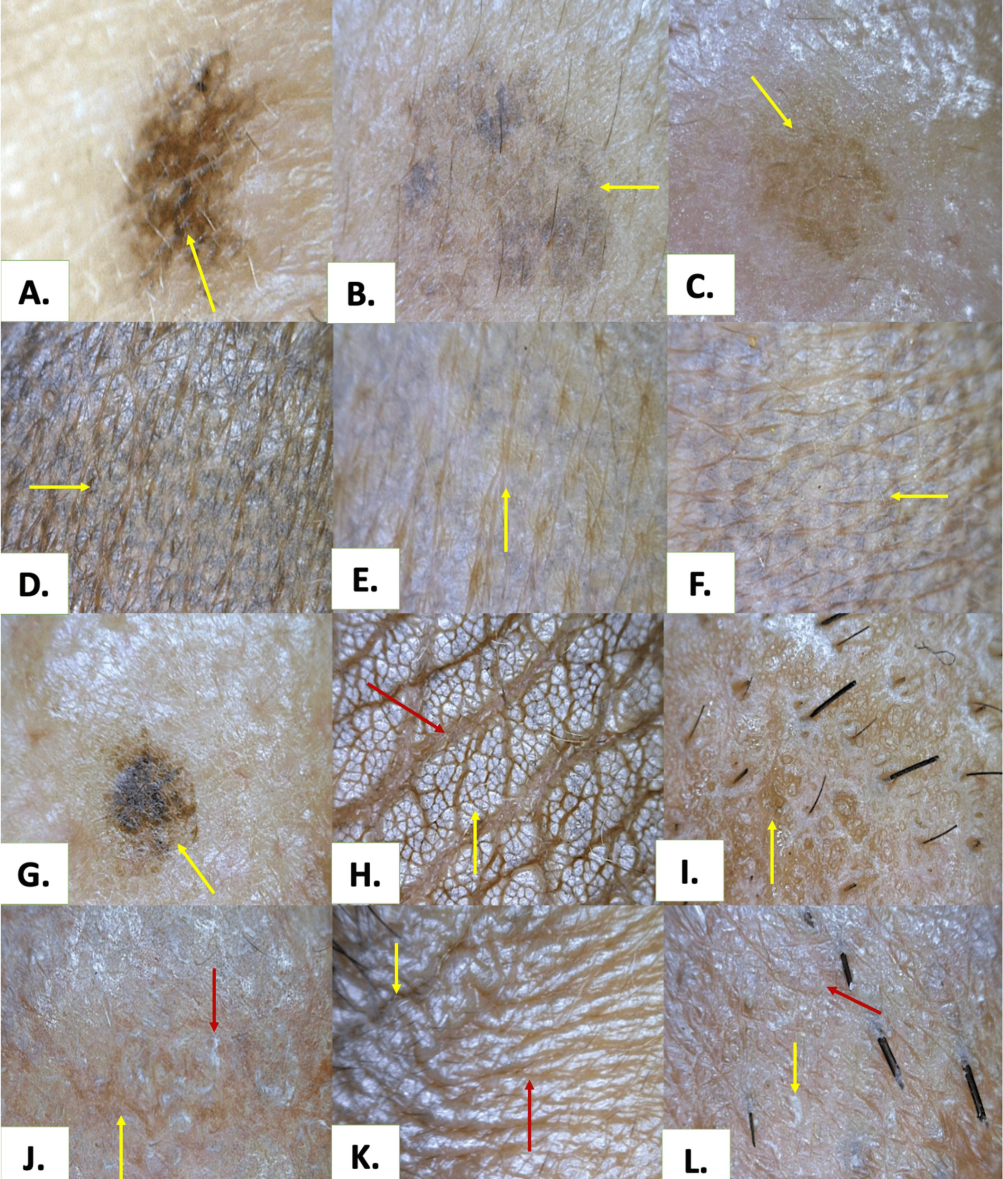
Common pigmentary dermoscopic patterns of various facial melanosis (polarised; 50x) A. Reticulo-globular pattern in melasma (yellow arrow); B. Blotches in melasma (yellow arrow); C. Brown structureless areas in melasma (yellow arrow); D. Reticular pattern in lichen planus pigmentosus (yellow arrow); E. Honeycombing in lichen planus pigmentosus (yellow arrow); F. Reticular pattern in fixed drug eruption (yellow arrow); G. Moth eaten appearance in lentigines (yellow arrow); H. Linear crista cutis (yellow arrow) and sulcus cutis (red arrow)in acanthosis nigricans; I. Cobblestone appearance in melasma with photoageing (yellow arrow); J. Telengiectasias (yellow arrow)with yellowish-white scales (red arrow) in topical steroid-damaged face; K. Grey structureless areas (yellow arrow) with sharp demarcation from surrounding skin (red arrow) of peri orbital hyperpigmentation; L. Erythema with scaling (yellow arrow) and brown structureless areas (red arrow) in Riehl's melanosis

**Figure 4:**
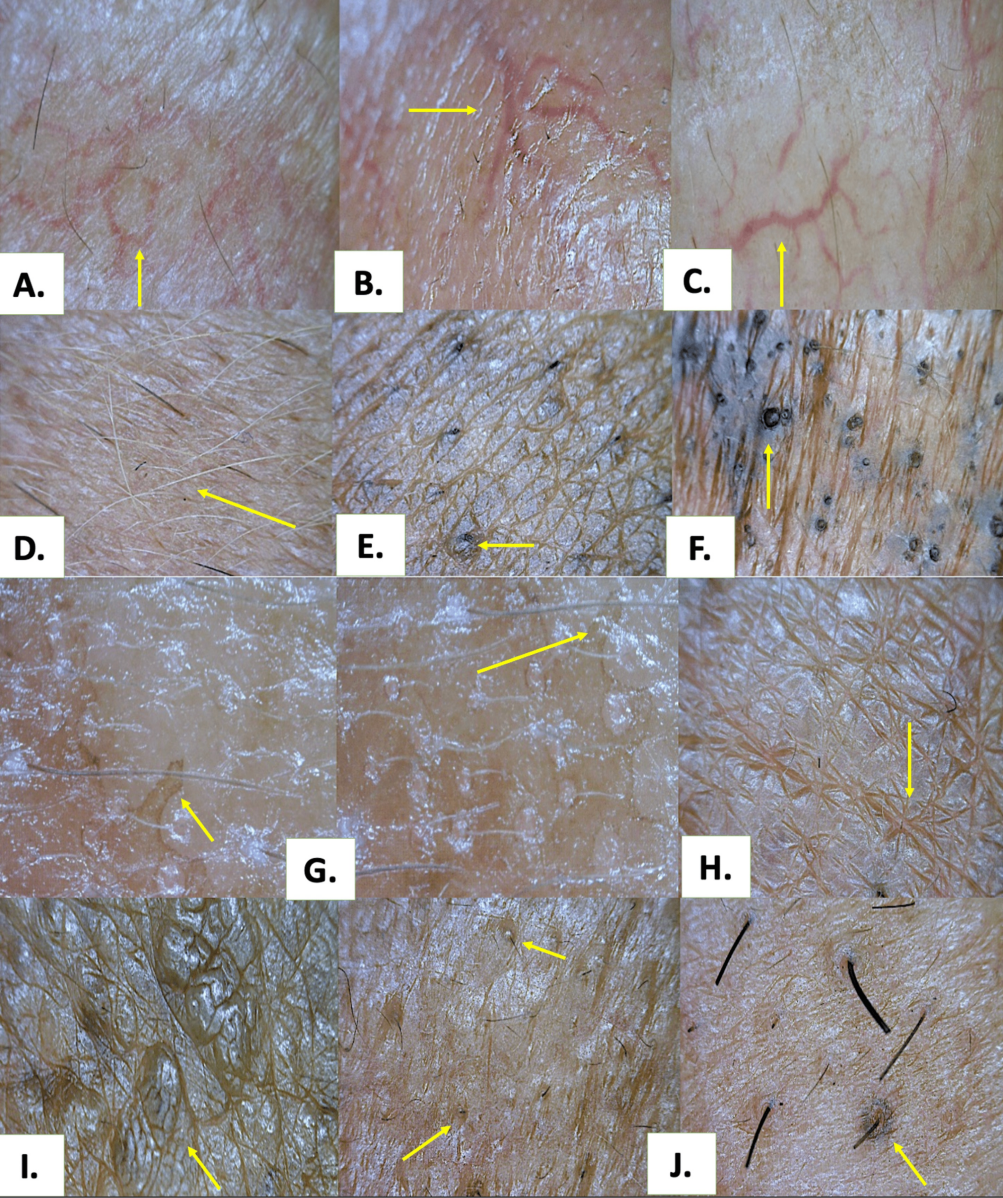
Vascular, appendageal and atypical dermoscopic patterns of various facial melanosis (polarised; 50x) A. Polygonal vessels in topical steroid-damaged face (yellow arrow); B. Arborizing vessels in topical steroid-damaged face (yellow arrow); C. Superficial dilated vessels in melasma with topical steroid-damaged face (yellow arrow); D. Leucotrichia in topical steroid-damaged face (yellow arrow); E. Perifollicular pigment deposition in lichen planus pigmentosus (yellow arrow); F. Patulous follicular plugs with post inflammatory hyperpigmentation in systemic lupus erythematous (yellow arrow); G. Pseudopodia in melasma (yellow arrow); H. Stellate furrowing in melasma with topical steroid-damaged face (yellow arrow); I. Cerebriform pattern in melasma with topical steroid-damaged face (yellow arrow); J. Perifollicular black halos in melasma (yellow arrow)

The most frequent dermoscopic pigmentary findings (Figure 3) were brown structureless areas (n = 107, 71.3%), blotches (n = 78, 52.0%), and brown-black globules (n = 78, 52.0%), followed by reticulo-globular (n = 68, 45.3%) and pseudo-reticular patterns (n = 58, 38.7%). Grey structureless areas were observed in 23 participants (15.3%), predominantly in dermal pigmentary disorders. The most common vascular feature was superficial dilated vessels, identified in 88 participants (58.7%). Appendageal alterations included follicular sparing (n = 40, 26.7%), hypertrichosis (n = 22, 14.7%), perifollicular pigment deposition (n = 16, 10.7%), and leucotrichia (n = 15, 10.0%). Infrequent atypical findings, including perifollicular black halos, pseudopodia, cerebriform patterns, and stellate furrowing, were observed in a limited number of cases, mainly across melasma and steroid-modified phenotypes (Figure 4).

The detailed distribution of dermoscopic features across individual diagnostic entities is presented in Table 3.

**Table 3:** Dermoscopic pigmentary, vascular, and appendageal patterns across facial melanosis (N = 150) Abbreviations: AN, acanthosis nigricans; CLD, chronic liver disease; EMFFEC, erythromelanosis follicularis faciei et colli; EPPB, erythrosis pigmentosa peribuccalis of Brocq; FDE, fixed drug eruption; LPP, lichen planus pigmentosus; PDL, pigmentary demarcation lines; PIH, post-inflammatory hyperpigmentation; POH, periorbital hyperpigmentation; SLE, systemic lupus erythematosus; SM, seborrhoeic melanosis; TSDF, topical steroid–damaged face; XP, xeroderma pigmentosum.

No.	Diagnosis (N, %)	Predominant Pigmentary Patterns n (%)	Vascular Patterns n (%)	Appendageal / Surface Features n (%)
STANDALONE CONDITIONS				
1	Melasma (37, 24.7)	Brown structureless 34 (91.9); blotches 25 (67.6); brown-black globules 23 (62.2); reticulo-globular 23 (62.2); pseudo-reticular 13 (35.1)	Superficial dilated vessels 24 (64.9)	Follicular sparing 24 (64.9)
2	LPP (9, 6.0)	Reticular 7 (77.8); pseudo-reticular 4 (44.4); black pigment dots 4 (44.4)	Superficial dilated vessels 3 (33.3)	Perifollicular pigment deposition 3 (33.3); follicular plugs 2 (22.2)
3	Nevus of Ota (6, 4.0)	Grey-blue dots 3 (50.0); brown-black globules 2 (33.3)	Superficial dilated vessels 1 (16.7)	—
4	Ashy dermatosis (4, 2.7)	Grey structureless areas 4 (100); faint reticular 4 (100); granular 2 (50.0)	Superficial dilated vessels 2 (50.0)	Perifollicular pigment deposition 4 (100)
5	Riehl’s melanosis (3, 2.0)	Yellowish-white scales 3 (100); brown structureless 2 (66.7)	Superficial dilated vessels 3 (100)	Follicular plugs 2 (66.7)
6	Hori’s nevus (3, 2.0)	Grey-blue dots 2 (66.7); brown-black globules 2 (66.7)	Superficial dilated vessels 1 (33.3)	—
7	Acanthosis nigricans (3, 2.0)	Linear crista & sulcus cutis 3 (100); acanthotic background 2 (66.7)	—	Perifollicular pigment deposition 3 (100); patulous follicles 2 (66.7)
8	POH (2, 1.3)	Brown structureless 1 (50.0); grey structureless 1 (50.0)	Superficial dilated vessels 2 (100)	—
9	PIH – varicella (2, 1.3)	Blotches 2 (100); brown/grey structureless 1 (50.0)	Superficial dilated vessels 2 (100)	Perifollicular pigment deposition 1 (50.0)
10	PIH – FDE (1, 0.7)	Pseudo-reticular 1 (100); reticulo-globular 1 (100)	Superficial dilated vessels 1 (100)	—
11	PIH – SLE (1, 0.7)	Black dots 1 (100); granular 1 (100); blotches 1 (100)	Superficial dilated vessels 1 (100)	Perifollicular pigment deposition 1 (100); leucotrichia 1 (100)
12	PIH – trauma (1, 0.7)	Brown structureless 1 (100); blotches 1 (100)	Superficial dilated vessels 1 (100)	—
13	Mucosal lentigines (1, 0.7)	Pseudo-reticular 1 (100)	Superficial dilated vessels 1 (100)	Yellowish-white scales 1 (100)
14	Nevus spilus (1, 0.7)	Moth-eaten border 1 (100); brown structureless 1 (100)	—	—
15	CLD-induced pigmentation (1, 0.7)	Brown + grey structureless 1 (100)	Superficial dilated vessels 1 (100)	—
16	Exogenous pigmentation (1, 0.7)	Black dots 1 (100); blotches 1 (100)	Superficial dilated vessels 1 (100)	—
17	EMFFEC (1, 0.7)	Brown structureless 1 (100)	Superficial dilated vessels 1 (100)	Perifollicular pigment deposition 1 (100)
18	Seborrhoeic melanosis (1, 0.7)	Yellowish-white scales 1 (100); mixed pigmentation 1 (100)	Superficial dilated vessels 1 (100)	Follicular plugs 1 (100)
19	Unilateral partial lentiginosis (1, 0.7)	Moth-eaten border 1 (100); coarse speckling 1 (100)	—	—
20	Xeroderma pigmentosum (1, 0.7)	Reticulo-globular 1 (100)	—	—
21	Lentigines (1, 0.7)	Moth-eaten border 1 (100); pseudo-reticular 1 (100)	—	—
OVERLAPPING CONDITIONS				
22	Melasma + TSDF (30, 20.0)	Brown structureless 29 (96.7); globules 21 (70.0); blotches 19 (63.3); pseudo-reticular 16 (53.3)	Superficial dilated 25 (83.3); arborizing 7 (23.3)	Hypertrichosis 14 (46.7); leucotrichia 10 (33.3)
23	Melasma + EPPB (9, 6.0)	Reticulo-globular 9 (100); brown/grey structureless 9 (100)	Superficial dilated vessels 7 (77.8)	Leucotrichia 1 (11.1)
24	Melasma + Lentigines (9, 6.0)	Brown-black globules 9 (100); moth-eaten borders 7 (77.8)	Superficial dilated vessels 4 (44.4)	—
25	Melasma + POH (4, 2.7)	Blotches 4 (100); reticulo-globular 4 (100)	Superficial dilated vessels 3 (75.0)	Follicular sparing 2 (50.0)
26	Melasma + PDL (3, 2.0)	Brown structureless 3 (100); grey structureless 2 (66.7)	Superficial dilated vessels 2 (66.7)	—
27	Melasma + TSDF + Lentigines (3, 2.0)	Pseudo-reticular 3 (100); globules 3 (100)	Superficial dilated vessels 2 (66.7)	Hypertrichosis 2 (66.7)
28	Melasma + Photoageing (2, 1.3)	Reticulo-globular 2 (100); annular/arcuate 1 (50.0)	Superficial dilated vessels 2 (100)	Yellowish-white scales 1 (50.0)
29	LPP + TSDF (1, 0.7)	Pseudo-reticular 1 (100); blotches 1 (100)	Superficial dilated vessels 1 (100)	Follicular plugs 1 (100)
30	Melasma + LPP + TSDF (1, 0.7)	Pseudo-reticular 1 (100); accentuated network 1 (100)	Superficial dilated vessels 1 (100)	Perifollicular pigment deposition 1 (100)
31	Melasma + Lentigines + EPPB + TSDF (1, 0.7)	Mixed globular + structureless 1 (100)	Arborizing vessels 1 (100)	Hypertrichosis 1 (100)
32	Melasma + Lentigines + Photoageing (1, 0.7)	Moth-eaten border 1 (100); coarse speckling 1 (100)	Superficial dilated vessels 1 (100)	Follicular sparing 1 (100)
33	Melasma + PDL + EPPB (1, 0.7)	Brown/grey structureless 1 (100)	Superficial dilated vessels 1 (100)	—
34	Melasma + TSDF + PDL (1, 0.7)	Mixed globular + structureless 1 (100)	Superficial dilated vessels 1 (100)	Hypertrichosis 1 (100)
35	PDL + POH (1, 0.7)	Grey structureless 1 (100); accentuated network 1 (100)	Superficial dilated vessels 1 (100)	—
36	PDL + POH + TSDF (1, 0.7)	Grey structureless 1 (100)	Superficial dilated vessels 1 (100)	Yellowish-white scales 1 (100)
37	POH + TSDF (1, 0.7)	Grey structureless 1 (100)	Superficial dilated vessels 1 (100)	Yellowish-white scales 1 (100)

Quality of life

The mean composite Skindex-16 score for the participants was 22.5 ± 13.6. Domain-wise analysis showed a mean symptom score of 7.44 ± 14.9, a mean emotional score of 50.7 ± 23.0, and a mean functioning score of 9.40 ± 15.5. Domain-wise scores for each condition are shown in Table 4.

**Table 4:** Quality of life scores (Skindex-16) in facial melanosis (N = 150) Values expressed as mean ± SD; higher scores indicate poorer quality of life. Abbreviations: AN, acanthosis nigricans; CLD, chronic liver disease; EMFFEC, erythromelanosis follicularis faciei et colli; EPPB, erythrosis pigmentosa peribuccalis of Brocq; FDE, fixed drug eruption; LPP, lichen planus pigmentosus; PDL, pigmentary demarcation lines; PIH, post-inflammatory hyperpigmentation; POH, periorbital hyperpigmentation; SD, standard deviation; SLE, systemic lupus erythematosus; SM, seborrhoeic melanosis; TSDF, topical steroid–damaged face; XP, xeroderma pigmentosum.

No.	Diagnosis	N	Composite Score	Symptom Score	Emotion Score	Functioning Score
STANDALONE CONDITIONS						
1	Melasma	37	15.5 ± 7.46	0.56 ± 2.81	42.5 ± 17.1	3.42 ± 8.22
2	LPP	9	26.3 ± 8.91	4.17 ± 8.07	61.4 ± 18.4	13.3 ± 15.2
3	Nevus of Ota	6	42.0 ± 8.15	0.00	90.9 ± 6.64	35.0 ± 20.1
4	Ashy dermatosis	4	24.7 ± 12.6	16.7 ± 14.0	44.0 ± 7.4	13.3 ± 18.3
5	Riehl’s melanosis	3	15.0 ± 4.84	11.1 ± 10.5	29.4 ± 12.2	4.44 ± 7.70
6	Hori’s nevus	3	28.1 ± 8.16	0.00	77.8 ± 14.6	6.67 ± 11.5
7	Acanthosis nigricans	3	25.3 ± 9.18	1.39 ± 2.41	69.0 ± 24.7	5.55 ± 6.94
8	POH	2	17.6 ± 14.8	8.34 ± 11.8	34.5 ± 18.5	10.0 ± 14.1
9	PIH (varicella)	2	15.1 ± 12.3	2.08 ± 2.95	38.1 ± 26.9	5.00 ± 7.07
10	PIH (FDE-recurrent)	1	36.4	12.5	66.7	30.0
11	PIH (SLE)	1	59.0	8.33	95.2	73.3
12	PIH (trauma)	1	0.00	0.00	23.8	0.00
13	Mucosal lentigines	1	22.2	0.00	66.7	0.00
14	Nevus spilus	1	24.0	0.00	61.9	10.0
15	CLD-induced pigmentation	1	29.4	0.00	71.4	16.7
16	Exogenous pigmentation	1	27.8	0.00	83.3	0.00
17	EMFFEC	1	15.7	4.17	42.9	0.00
18	Seborrhoeic melanosis	1	5.55	0.00	16.7	0.00
19	Unilateral partial lentiginosis	1	40.2	0.00	90.5	30.0
20	Xeroderma pigmentosum	1	10.0	0.00	30.0	0.00
21	Lentigines	1	31.6	0.00	71.4	23.3
OVERLAPPING CONDITIONS						
22	Melasma + TSDF	30	27.5 ± 14.2	21.8 ± 21.3	50.7 ± 19.5	9.89 ± 13.0
23	Melasma + EPPB	9	17.9 ± 12.9	1.85 ± 5.56	47.5 ± 29.0	4.44 ± 7.99
24	Melasma + Lentigines	9	11.7 ± 6.78	0.00	34.7 ± 20.1	0.37 ± 1.11
25	Melasma + POH	4	22.7 ± 9.45	0.00	61.3 ± 29.6	6.67 ± 3.85
26	Melasma + PDL	3	24.2 ± 15.6	0.00	58.1 ± 22.0	14.4 ± 25.0
27	Melasma + TSDF + Lentigines	3	38.6 ± 23.6	31.9 ± 22.0	59.5 ± 21.4	24.4 ± 22.7
28	Melasma + Photoageing	2	29.2 ± 32.3	10.4 ± 14.7	40.5 ± 30.3	36.7 ± 51.9
29	LPP + TSDF	1	59.4	41.7	80.0	56.7
30	Melasma + LPP + TSDF	1	33.5	33.3	57.1	10.0
31	Melasma + Lentigines + EPPB + TSDF	1	31.1	20.8	69.0	3.33
32	Melasma + Lentigines + Photoageing	1	17.0	0.00	47.6	3.33
33	Melasma + PDL + EPPB	1	11.1	0.00	33.3	0.00
34	Melasma + TSDF + PDL	1	35.2	25.0	73.8	6.67
35	PDL + POH	1	6.35	0.00	19.1	0.00
36	PDL + POH + TSDF	1	6.35	0.00	19.1	0.00
37	POH + TSDF	1	6.35	0.00	19.1	0.00

## Discussion

Facial melanosis in skin of color represents not a single disease entity but a phenotypic convergence of melanocyte hyper-reactivity, environmental exposure, and cultural practice, unfolding on the most socially visible surface of the body. This study sought to interrogate that convergence: Who are the affected individuals? What disorders dominate? How do these conditions look under dermoscopy? And crucially, how do they feel about living with them?

Demographic and clinical context: who bears the burden?

The marked female predominance and peak involvement in the third to fourth decades observed in this study reflect a consistent epidemiological signature reported across Indian and South Asian studies [9-11]. This demographic clustering raises an immediate question: “Is facial melanosis hormonally driven, socially conditioned, or both?” The high frequency of melasma, either as a standalone or overlapping diagnosis, alongside pregnancy-related exacerbation and oral contraceptive exposure, supports a hormonal substrate [12-14]. Yet, the parallel prominence of TSDF and cosmetic use underscores the role of sociocultural pressures surrounding complexion, particularly in women of reproductive age.

In this study, homemakers formed the largest occupational group. This distribution likely reflects the demographic profile and healthcare-seeking behavior of the study population rather than a direct occupational risk. Although many participants were engaged in predominantly indoor roles, they may still encounter contributory factors such as intermittent sun exposure and cosmetic use. Such findings underscore the paradox that even those working indoors are not spared: daily rituals of cosmetic application, cultural practices of skin lightening, and indirect sun exposure conspire with equal force as outdoor labor. 

Hypothyroidism emerged as the most common comorbid condition, predominantly among participants with melasma, consistent with observations from previous Indian studies and suggesting a potential endocrine contribution to melanogenesis. A positive family history of pigmentation disorders was noted in 21.3% of participants, chiefly in those with melasma, with clustering among first-degree female relatives, supporting a genetic predisposition possibly modulated by hormonal factors [9,11].

The predominance of Fitzpatrick skin types IV and V situates this study firmly within the biological framework of skin of color. Here, melanin functions paradoxically, as photoprotective armor and as a liability, rendering pigmentary responses exaggerated, persistent, and visually conspicuous. This duality explains not only the frequency of facial melanosis in darker phototypes but also its chronicity and therapeutic resistance [15-17].

Diagnostic spectrum: why does overlap dominate?

A key strength of this study lies in its recognition that facial melanosis rarely respects diagnostic silos. While 22 distinct conditions were identified, a substantial proportion of participants exhibited overlapping or composite diagnoses, most commonly melasma coexisting with TSDF, lentigines, or EPPB. This raises a critical conceptual question: “Are these overlaps coincidental, or do they represent sequential pathogenic layering?”

The data favor the latter. Chronic ultraviolet exposure, steroid misuse, and low-grade inflammation likely remodel the cutaneous microenvironment, allowing multiple pigmentary pathways to coexist within the same facial unit. By explicitly separating standalone from overlapping entities and then reconsolidating them into broader clinical categories, this study avoids diagnostic fragmentation while preserving real-world complexity, an approach particularly relevant in skin of color, where hybrid phenotypes are common yet underreported.

Melasma emerged as the most common diagnosis, likely reflecting the convergence of genetic susceptibility, heightened melanocyte reactivity in skin of color, and chronic ultraviolet exposure acting on hormonally responsive facial skin. Additional amplification by pregnancy-related hormonal shifts, cosmetic use, and inadvertent topical corticosteroid exposure may further explain its predominance within this population.

Dermoscopy: what lies beneath the color?

Across the study, brown structureless areas, blotches, and brown-black globules dominated, reflecting epidermal or mixed melanin deposition, particularly in melasma and its overlaps. In contrast, slate-grey and bluish tones, coupled with grey structureless areas and granular patterns, were preferentially associated with dermal pigmentary disorders such as LPP and ashy dermatosis. These findings reaffirm that in skin of color, color is histology made visible [18-20].

Vascular patterns added another dimension. The frequent identification of superficial dilated vessels, especially in TSDF and melasma, raises an important mechanistic consideration: “Is vascular change a passenger or a driver of pigmentation?” Emerging evidence suggests that angiogenic mediators and vascular instability may amplify melanogenesis, particularly in steroid-modified skin [12-14,21].

Appendageal features, including follicular sparing, perifollicular pigmentation, hypertrichosis, and leucotrichia, further refined diagnostic specificity, highlighting how follicles act as both sanctuaries and targets in pigmentary disease.

Notably, this study documented several infrequent and atypical dermoscopic features that are rarely described in facial melanosis and represent a distinctive contribution of our work.

Quality of life: where does the true burden lie?

Perhaps the most sobering insight from this study arises from the quality of life analysis. Despite low symptom scores, the emotional domain of Skindex-16 was disproportionately elevated, underscoring a central truth: facial melanosis wounds identity more than it irritates skin. The mean composite score (22.5 ± 13.6) aligns with prior Indian data [22], yet the emotional scores in this study were notably high, reflecting distress, self-consciousness, and altered self-perception. 

Conditions with chronicity, dermal depth, or iatrogenic stigma, particularly TSDF-associated pigmentation and LPP, demonstrated the highest emotional burden. Importantly, certain solitary but visually striking conditions also showed high scores, reminding clinicians that rarity does not equate to insignificance. These findings caution against trivializing pigmentary disorders as cosmetic, especially in cultures where facial appearance carries profound social meaning.

Strengths, limitations, and future directions

The principal strength of this study lies in its integrated framework, simultaneously addressing clinical morphology, dermoscopic architecture, and psychosocial impact in a large, skin-of-color-dominant participant group. The explicit documentation of overlapping diagnoses and appendageal features adds nuance rarely captured in prior literature.

Limitations include the cross-sectional design, which precludes temporal inference, and small numbers within certain rare entities, warranting cautious interpretation of condition-specific quality of life scores. As this was a descriptive study, age-, gender-, and occupation-related associations were not formally analyzed, and these findings are interpreted as distributions rather than determinants. Histopathological correlation, while not feasible in all cases, would further enrich dermoscopic interpretation.

## Conclusions

Facial melanosis in skin of color extends beyond a mere disorder of pigmentation, reflecting issues of visibility, self-perception, and lived experience. In keeping with existing literature, melasma emerged as the most common diagnosis with a clear female preponderance, while dermatoscopic evaluation not only confirmed established patterns but also identified several atypical features unique to this study. The inclusion of quality of life assessment adds a critical patient-centered dimension, collectively advancing understanding of facial melanosis beyond surface color to encompass both its biological heterogeneity and psychosocial burden.

In an era where dermatology is increasingly procedural and algorithmic, this work recenters attention on pattern recognition, context, and lived experience. For clinicians, it offers a dermoscopic vocabulary tailored to skin of color. For researchers, it highlights the need to study overlap rather than purity. And for patients, it validates that facial pigmentation is not superficial; it is deeply human. Facial melanosis in skin of color is not merely pigment. It is memory, identity, and visibility. This study brings us one step closer to seeing it clearly. 

## Figures and Tables

**Figure 5:**
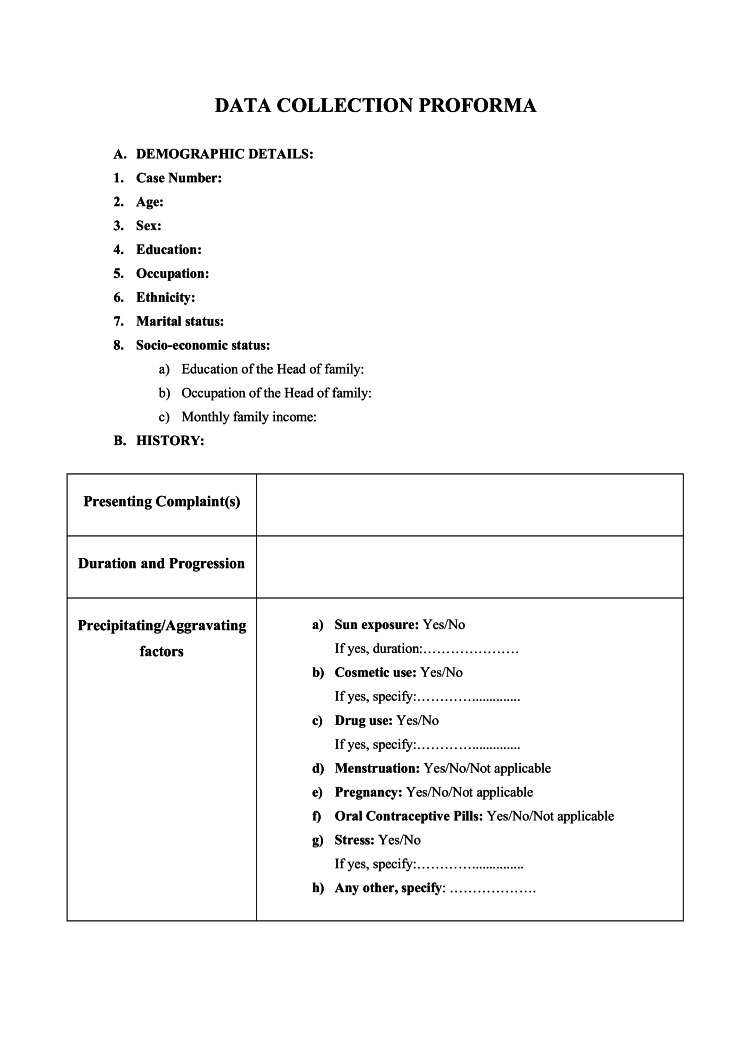
Part 1 of the questionnaire

**Figure 6:**
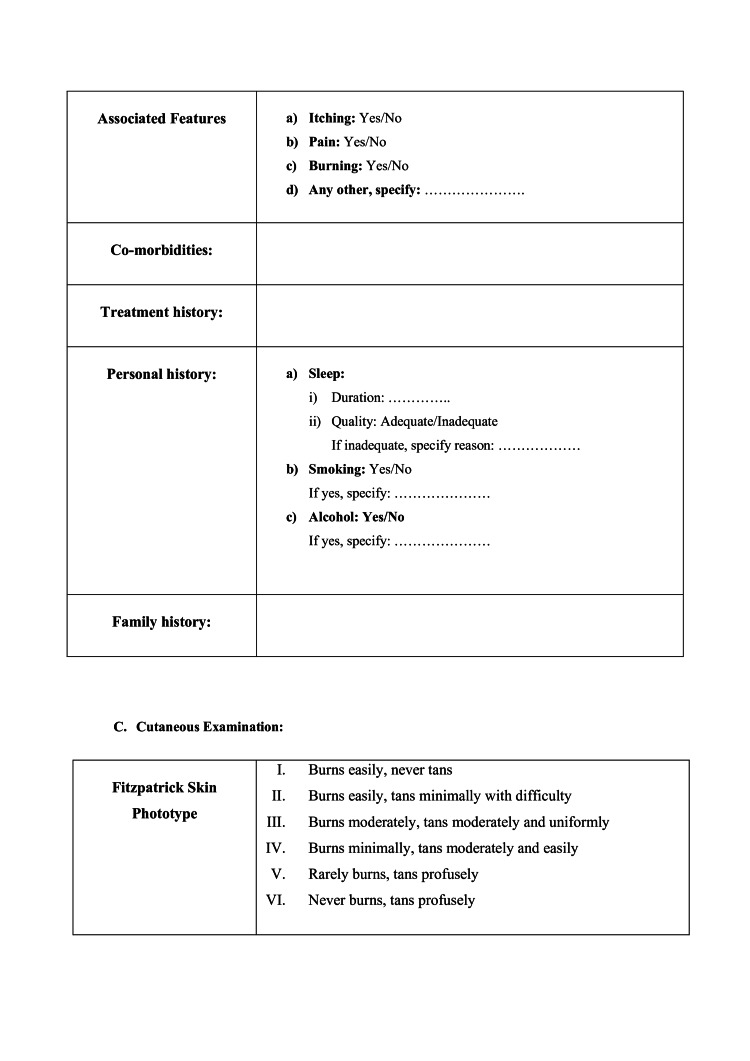
Part 2 of the questionnaire

**Figure 7:**
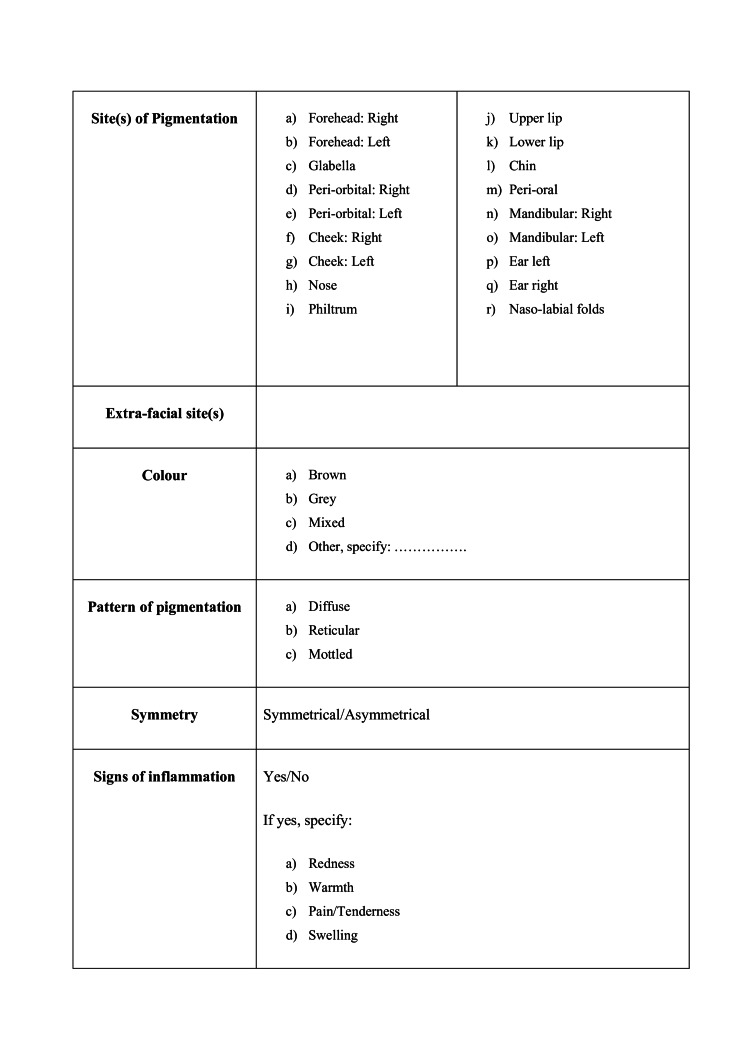
Part 3 of the questionnaire

**Figure 8:**
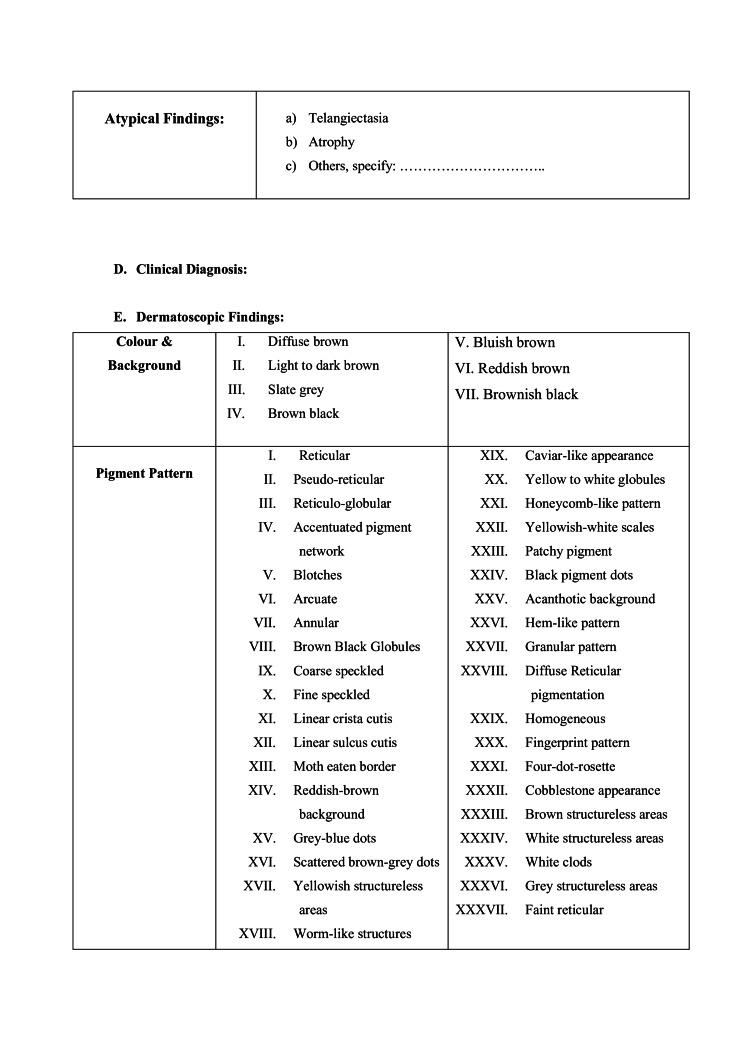
Part 4 of the questionnaire

**Figure 9:**
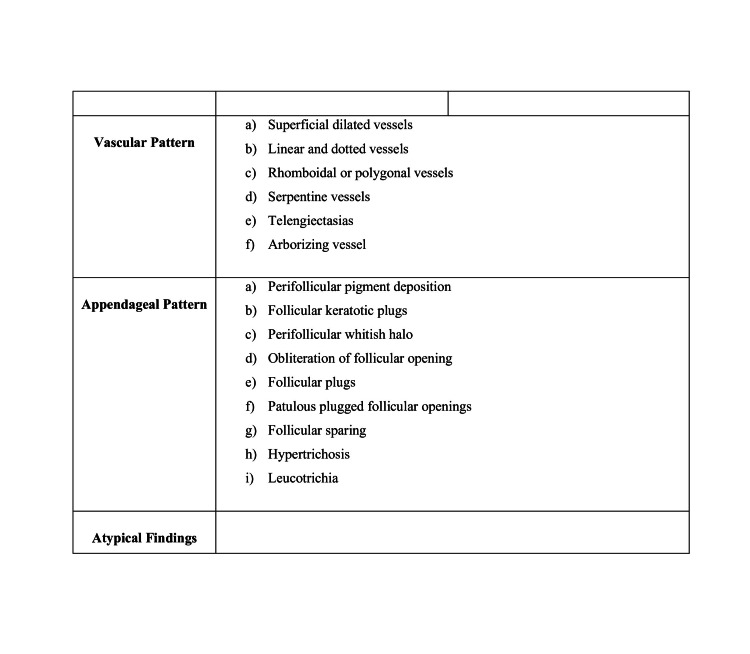
Part 5 of the questionnaire

## Appendices

Appendix A
